# Risk assessment according to IPSS-M is superior to AML ELN risk classification in MDS/AML overlap patients defined by ICC

**DOI:** 10.1038/s41375-023-02004-w

**Published:** 2023-08-12

**Authors:** Sandra Huber, Constance Baer, Stephan Hutter, Frank Dicker, Irene Fuhrmann, Manja Meggendorfer, Christian Pohlkamp, Wolfgang Kern, Torsten Haferlach, Claudia Haferlach, Gregor Hoermann

**Affiliations:** MLL Munich Leukemia Laboratory, Max-Lebsche-Platz 31, 81377 Munich, Germany

**Keywords:** Acute myeloid leukaemia, Myelodysplastic syndrome

## To the Editor:

MDS/AML overlap has recently been introduced as novel myeloid disease entity by the International Consensus Classification (ICC) with 10–19% blasts in the absence of AML-defining recurrent genetic abnormalities, acknowledging the biologic continuum between MDS and AML [1]. MDS/AML is not recognized as separate entity according to the 5th edition of the WHO classification (WHO 2022; [2]) where it largely overlaps with MDS with increased blasts 2 (MDS-IB2). A main argument of the ICC in favor of the MDS/AML category has been a potential eligibility of these patients for clinical trials of either MDS or AML [3].

Risk assessment is often considered for clinical trial inclusion criteria. In MDS patients, risk stratification is based on the revised International Prognostic Scoring System (IPSS-R; [4]) and the recently published IPSS-M which incorporates molecular genetics in addition to cytogenetics and clinical parameters [5, 6]. Regarding the IPSS-M, a continuous patient-specific risk score is grouped into six risk categories: very low (VL), low (L), moderate low (ML), moderate high (MH), high (H) and very high (VH). In contrast, AML patients are risk stratified according to the European Leukemia Net (ELN) 2022 system classifying AML patients into favorable, intermediate and adverse risk groups depending on the underlying genetics [7]. There are no guidelines or data available how MDS/AML patients should be risk stratified. We therefore asked whether MDS- and/or AML-based risk stratification according to IPSS-M and ELN 2022 were appropriate for MDS/AML patients.

We identified 137 patients with MDS/AML according to ICC in a cohort of 1,451 patients with non-therapy related myeloid neoplasm analyzed by whole genome and transcriptome sequencing (WGS: median coverage 100x; WTS: median yield 50 million reads; Supplementary Methods; [8, 9]). Bona fide MDS patients according to WHO 2017 (*n* = 626; [8]) and AML patients according to WHO 2022 (*n* = 686; [9]) were used for comparison (Supplementary Tables S1–S3). We restrained from exclusion of MDS/AML patients according to ICC in the comparison cohorts to avoid a selection bias resulting in a partial overlap of 116 patients with the MDS and none with the AML cohort. All patients gave their written informed consent and the study was approved by the laboratory´s institutional review board and conducted according to the Declaration of Helsinki. Analyses for overall survival (OS) were performed according to Kaplan-Meier and compared using two-sided log rank tests. The OS was calculated as time from diagnosis to death or last follow-up. To assess the correlation between risk scores and real outcomes we used the Harrell’s concordance index (c-index [10]). All results were considered significant at *p* < 0.05.

The MDS/AML cohort comprised 58 (42%) female and 79 (58%) male cases with a median age of 74 [32–91] years and a median follow-up of 9.5 years (Supplementary Table S1). Median OS was 2.1 years. First, MDS/AML patients (*n* = 137) were subclassified as proposed by ICC (Supplementary Fig. S1A, Table S1) into MDS/AML with mutated *TP53* (*n* = 19; 14%), with myelodysplasia-related (MR) gene mutations (MR muts; *n* = 99; 72%) or MR cytogenetic abnormalities (MR cyto; *n* = 6; 4%), or not otherwise specified (NOS; *n* = 13; 10%). MDS/AML subgroups showed significant differences in OS (except between MR cyto vs. MR muts with 1.3 and 2.3 years), with MDS/AML-*TP53* having the shortest and MDS/AML-NOS the longest median OS (0.8 and 8.2 years, respectively; Supplementary Fig. S1A). This is in line with a previous study by Lee et al. analyzing 173 MDS/AML patients that additionally found OS differences between MR subgroups [11]. As expected, MDS/AML patients were mainly composed of MDS-IB2 (114/137; 83%) according to WHO 2022. In addition, MDS/AML-*TP53* corresponded to MDS-bi*TP53* based on WHO 2022 (*n* = 19; Supplementary Table S4), while the remaining 4 MDS/AML patients were classified as AML based on WHO 2022 as harboring specific *MECOM*- (*n* = 3) or *KMT2A*- (*n* = 1) rearrangements that were not considered as recurrent defining genetic abnormalities according to ICC but according to WHO 2022 (Supplementary Fig. S1B).

**Fig. 1:**
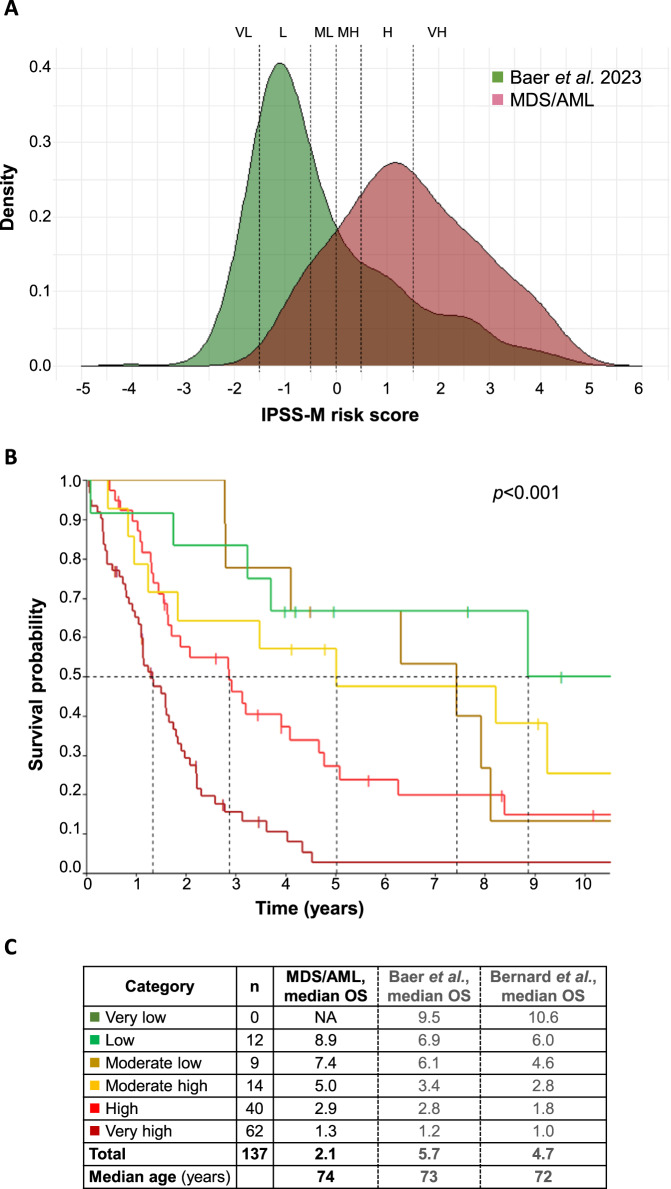
Risk-stratification of MDS/AML patients according to IPSS-M. **A** Density plot of IPSS-M risk scores calculated for MDS/AML patients (red; *n* = 137) and for the bona fide MDS cohort ([8]; green; *n* = 626). VL: very low, L: low, ML: moderate low, MH: moderate high, H: high, VH: very high. **B** Overall survival (OS) of the MDS/AML cohort (*n* = 137) according to IPSS-M risk categories: low/ green (*n* = 12), moderate low/ brown (*n* = 9), moderate high/ yellow (*n* = 14), high/ red (*n* = 40), very high/ dark red (*n* = 62); dotted line: median OS; see also **C**. **C** Survival data of the IPSS-M risk categories of different cohorts; OS overall survival, NA not available, n number of samples of MDS/AML cohort.

We then focused on the MDS-based risk prediction using the IPSS-M. As expected for an MDS/AML cohort, resulting categories showed a clear skewing towards high-risk groups (45% VH, 29% H, 10% MH, 7% ML, 9% L and 0% VL) compared to a bona fide MDS cohort [8] used as control (14%, 12%, 7%, 11%, 41% and 15%, respectively) (Fig. 1A). Importantly, a clear separation of MDS/AML patients assigned to the IPSS-M risk groups (*p* < 0.001; Fig. 1B) was found with respect to OS. Notably, the OS of the respective risk groups was well comparable to the bona fide MDS cohort ([8]; Fig. 1C; Supplementary Fig. S2A). To correct for a potential bias due to overlapping samples, we also contrasted MDS/AML patients to a down-sampled sex-matched MDS cohort (*n* = 137, excluding overlapping MDS/AML cases) as well as an independently published unselected MDS cohort (*n* = 2701; [5]) and observed well comparable results (Fig. 1C; Supplementary Fig. S2B). The fit of the IPSS-M models reflected by the c-index was similar for the MDS/AML cohort (0.7125), the control MDS cohort ([8]; 0.7155) and the down-sampled sex-matched MDS group (0.7166).

**Fig. 2:**
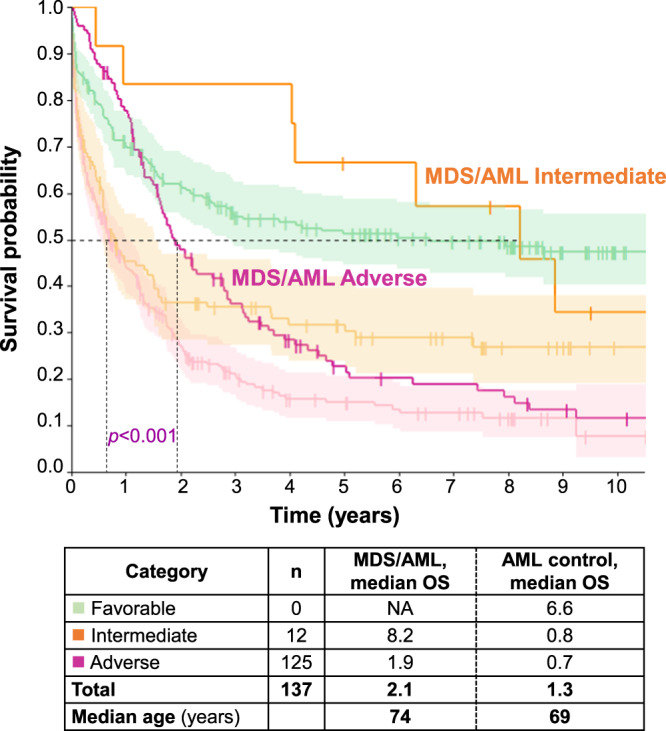
Risk-stratification of MDS/AML patients according to ELN 2022 guidelines. Overall survival (OS) according to ELN 2022 risk categories of the MDS/AML cohort (*n* = 137; orange line: intermediate risk; purple line: adverse risk) and the AML control group (*n* = 686; 95% confidence interval included for the different risk categories: favorable/light green, intermediate/light orange, adverse/light purple). NA not available.

Next, we grouped our MDS/AML patients according to AML-based risk classification using ELN 2022 guidelines. No MDS/AML patient fulfilled criteria for the favorable ELN risk group per definition. Notably, only 9% (12/137) were classified as intermediate risk, and the vast majority (91%; 125/137) as adverse risk. Adverse risk classification was primarily driven due to MR-associated gene mutations (99/125; 79%), *TP53* mutations (19/125; 15%) and/or MR-associated cytogenetic aberrations (36/125; 29%). Within MDS/AML, the intermediate risk group still showed longer OS than the adverse risk group (median OS: 8.2 vs. 1.9; *p* = 0.009). However, the survival of MDS/AML patients substantially differed from a bona fide AML control cohort (Fig. 2). In particular, the OS of MDS/AML patients classified as adverse risk according to ELN 2022 was significantly and clinically meaningfully longer than the corresponding adverse risk AML patients (median OS: 1.9 vs. 0.7; *p* < 0.001). Likewise, the OS of MDS/AML patients classified as intermediate risk according to ELN 2022 was also longer than the corresponding intermediate risk AML patients (median OS: 8.2 vs. 0.8; *p* = 0.057) (Fig. 2).

In summary, we confirmed the prognostic significance within MDS/AML entities according to ICC, in particular MDS/AML with *TP53*. We found that for MDS/AML patients, MDS-based risk assessment according to IPSS-M is fully applicable with comparable OS data to a real-world MDS cohort despite a skewing towards high-risk categories. In contrast, AML-based risk classification according to ELN 2022 guidelines is not applicable for MDS/AML. The classification of nearly all patients as adverse risk due to their MR-associated genetic profile is not meaningful—in particular as it is not supported by OS data. If MDS/AML patients were to be included into AML studies, development of a specific risk assessment for MDS/AML other than ELN 2022 would be needed.

Limitations of our study include the retrospective design, the non-uniform treatment, and the low number of patients in some of the IPSS-M risk groups. We cannot exclude that an AML-specific treatment of MDS/AML patients would affect the risk stratification as the number of intensively treated patients in our cohort was too small for subgroup analysis. In this regard, it has to be noted that the ELN risk stratification is meant to be applied to intensively treated patients. The performance of the ELN risk stratification—or a modified version of it—in intensively treated MDS/AML patients remains to be studied. However, the substantially better survival of MDS/AML patients compared to adverse risk AML despite more intensive therapy in the latter and higher age in the former (74 vs. 69 years in our cohort) raises substantial concerns about a potential justification of a general inclusion of MDS/AML patients in a clinical trial designed for adverse risk AML. Our data clearly support the WHO that decided not to introduce this MDS/AML category arguing that this may lead to the risk of overtreatment in some patients [2]. Neither the WHO classification nor our data argue against an individual decision for a trial enrolment in patients with MDS-IB2, in particular in young patients. However, the general concept of MDS/AML as defined by the ICC remains to be proven.

Taken together, the value of another arbitrary blast cell cut-off remains questionable. One could argue that blast cell thresholds remain inevitable in the field of MDS and AML. In contrast, we previously showed that a genetic-based MDS subclassification better reflects biology than blast counts and that the latter is rather a sequela of the former than an independent biological category [12]. This concept has the potential to improve outcome prediction and individual treatment choice beyond blast cell counting. Thus, clinical trial designs for MDS, MDS/AML and AML should evolve from arbitrary blast cell thresholds to the consideration of genetic subtypes and progression markers. This is further supported by Zeidan et al. emphasizing that treatment decisions need to include multidimensional assessment of the clinical history, symptom burden and the genetic characteristics of the disease [13]. Evaluating the suitability of a patient for certain treatments by these criteria is the rationale for clinical trial enrollments on a personalized/ individualized/ patient-specific level in the era of personalized medicine.

### Supplementary information

Supplementary Material

## Data Availability

The datasets generated during and/or analyzed during the current study are available from the corresponding author on reasonable request.
