# Supplementary material for: Risk assessment according to IPSS-M is superior to AML ELN risk classification in MDS/AML overlap patients defined by ICC
Source: Leukemia. 2023 Aug 12;37(10):2138–41. doi: 10.1038/s41375-023-02004-w (PMC10539168; doi:10.1038/s41375-023-02004-w)
Supplement: Supplementary file 1 — Supplementary Material [file 41375_2023_2004_MOESM1_ESM.pdf]

Supplementary Material

Supplementary Methods

Patients cohort and samples

All MDS/AML samples were sent to the MLL Munich Leukemia Laboratory between 09/2000 and 07/2009. Diagnoses (from peripheral blood and bone marrow) were made based on cytomorphology, cytogenetics and molecular genetics as previously published [1-3].

Whole genome and whole transcriptome sequencing (WGS, WTS)

WGS and WTS analysis were performed for all patients. For WGS, total genomic DNA was extracted from lysed cell pellet of bone marrow or peripheral blood using the MagNA Pure 96 with DNA and Viral Nucleic Acid Large Volume Kit and Cellular RNA Large Volume Kit (Roche, Basel, Switzerland). Library preparation and sequencing as well as calling and filtering of single nucleotide variants, structural variants and somatic copy number variations (CNVs) were performed as previously described [4, 5]. Copy neutral loss of heterozygosity (CN-LOH) was assessed using HadoopCNV [6]. WTS was performed as described therein [7]. Fusion calling was performed with Manta (v0.29.0) [8], Arriba (v1.2.0) [9] and STAR-Fusion (v1.9.0) [10].

Mutational analysis

Mutational data was retrieved from WGS data or during routine work-up. Structural variants/ fusions were analyzed by combining routine cytogenetics (encompassing chromosome banding analyses and FISH) and WTS.

Statistical analysis

For statistical analyses R version 4.2.2 (R Foundation for Statistical Computing, Vienna, Austria) with the survival and survminer packages was used.

Supplementary Results

Supplementary Tables and Figures

Table S1. MDS/AML cohort overview

**Characteristics**	**MDS/AML cases (n = 137)**
**Age** (years; median [range])	74 [32-91]
**Sex** (female / male)	58 (42%) / 79 (58%)
**Bone marrow blast count** (%; median [range])	13 [10-19]
**Karyotype** (normal / aberrant)	71 (52%) / 66 (48%)
**IPSS-R cytogenetic risk group**	**Number of samples (%)**
Very poor	24 (17)
Poor	9 (7)
Intermediate	22 (16)
Good	73 (53)
Very good	9 (7)
**ICC diagnosis**	
MDS/AML with mutated *TP53* (MDS/AML-*TP53*)	19 (14)
MDS/AML with MR gene mutations (MDS/AML-MR mut)	99 (72)
MDS/AML with MR cytogenetic abnormalities (MDS/AML-MR cyto)	6 (4)
MDS/AML, not otherwise specified (MDS/AML-NOS)	13 (10)
**Clinical data**	
Treatment data - availability	127 (93)
Intensive chemotherapy	18 (14)
Allogeneic HSCT	10 (8)
Not intensive chemotherapy	45 (35)
Supportive treatment	29 (23)
None	35 (28)
Response data - availability	104 (76)
CR reached	13 (13)
Progress to AML	45 (33)

MR: myelodysplasia-related; HSCT: hematopoietic stem cell transplantation; CR: complete remission.

Table S2. MDS cohort overview (without overlapping 116 MDS/AML cases)

**Characteristics**	**MDS cases (n = 510)**
**Age** (years; median [range])	73 [23-85]
**Sex** (female / male)	214 (42%) / 296 (58%)
**Bone marrow blast count** (%; median [range])	3 [0-19]
**Karyotype** (normal / aberrant)	310 (61%) / 200 (39%)
**IPSS-R cytogenetic risk group**	**Number of samples (%)**
Very poor	20 (4)
Poor	18 (3)
Intermediate	39 (8)
Good	402 (79)
Very good	31 (6)
**WHO 2017 diagnosis**	
MDS with single lineage dysplasia (MDS-SLD)	20 (4)
MDS with multilineage dysplasia (MDS-MLD)	97 (19)
MDS with single lineage dysplasia with ring sideroblasts (MDS-RS-SLD)	47 (9)
MDS with multilineage dysplasia with ring sideroblasts (MDS-RS-MLD)	128 (25)
MDS with isolated del(5q) (MDS 5q-)	87 (17)
MDS with excess blasts (MDS-EB-1)	119 (23)
MDS with excess blasts (MDS-EB-2)	12 (3)
**Clinical data**	
Treatment data - availability	497 (97)
Intensive chemotherapy	26 (5)
Allogeneic HSCT	21 (4)
Not intensive chemotherapy	123 (25)
Supportive treatment	192 (39)
None	137 (27)
Response data - availability	399 (78)
CR reached	26 (7)
Progress to AML	48 (9)

HSCT: hematopoietic stem cell transplantation; CR: complete remission.

Table S3. AML cohort overview

Characteristics	AML cases (n = 686)
**Age** (years; median [range])	69 [2-93]
**Sex** (female / male)	306 (45%) / 380 (55%)
**Bone marrow blast count** (%; median [range])	66 [1-99]
**Karyotype** (normal / aberrant)	268 (39%) / 418 (61%)
**WHO 2022 diagnosis**	**Number of samples (%)**
AML with *RUNX1::RUNX1T1* fusion	41 (6)
AML with *CBFB::MYH11* fusion	45 (7)
AML with *DEK::NUP214* fusion	15 (2)
AML with *KMT2A* rearrangement	44 (6)
AML with *MECOM* rearrangement	64 (9)
AML with *NUP98* rearrangement	5 (1)
AML with *NPM1* mutation	166 (24)
AML with *CEBPA* mutation	61 (9)
AML-MR	208 (30)
AML with other defined genetic alterations	1 (0)
AML defined by differentiation	36 (5)
**Clinical data**	
Treatment data - availability	597 (87)
Intensive chemotherapy	412 (69)
Allogeneic HSCT	156 (38)
Not intensive chemotherapy	108 (18)
Supportive treatment	66 (11)
Unspecified	11 (2)
Response data - availability	586 (85)
CR reached	320 (55)
Post-MDS or MDS/MPN *	34 (5)
Prior MDS or MDS/MPN treatment data – availability	29 (85)
Not intensive chemotherapy	8 (28)
Supportive treatment	11 (38)
None	10 (34)

MR: myelodysplasia-related; HSCT: hematopoietic stem cell transplantation; CR: complete remission.
* Prior MDS or MDS/MPN treatment did not impact on survival analysis of the AML cohort stratified for ELN risk categories.

Table S4. Characteristics of cases within *TP53* mutated entities

Characteristics	MDS/AML-*TP53* cases (n=19)	MDS-*TP53* cases* (n=15)	AML-*TP53* cases (n=48)
Complex karyotype	19; 100%	14; 93%	44; 92%
TP53 monoallelic	0; 0%	0 (per definitionem)	2; 4%
TP53 biallelic	19; 100%	15; 100%	46; 96%
Two mutations	3	5**	11
Mutation + deletion	8	6	24
Mutation + CN-LOH	8	4	11
Median *TP53* VAF % (range)	60 (17-93)	40 (4-96)	71 (20-99)

CN-LOH: Copy neutral loss of heterozygosity; VAF: Variant allelic frequency; * without overlapping 19 MDS/AML cases; ** including 2 cases with both *TP53* VAFs <10% (fulfilling WHO 2022 entity criteria, but not ICC entity criteria)

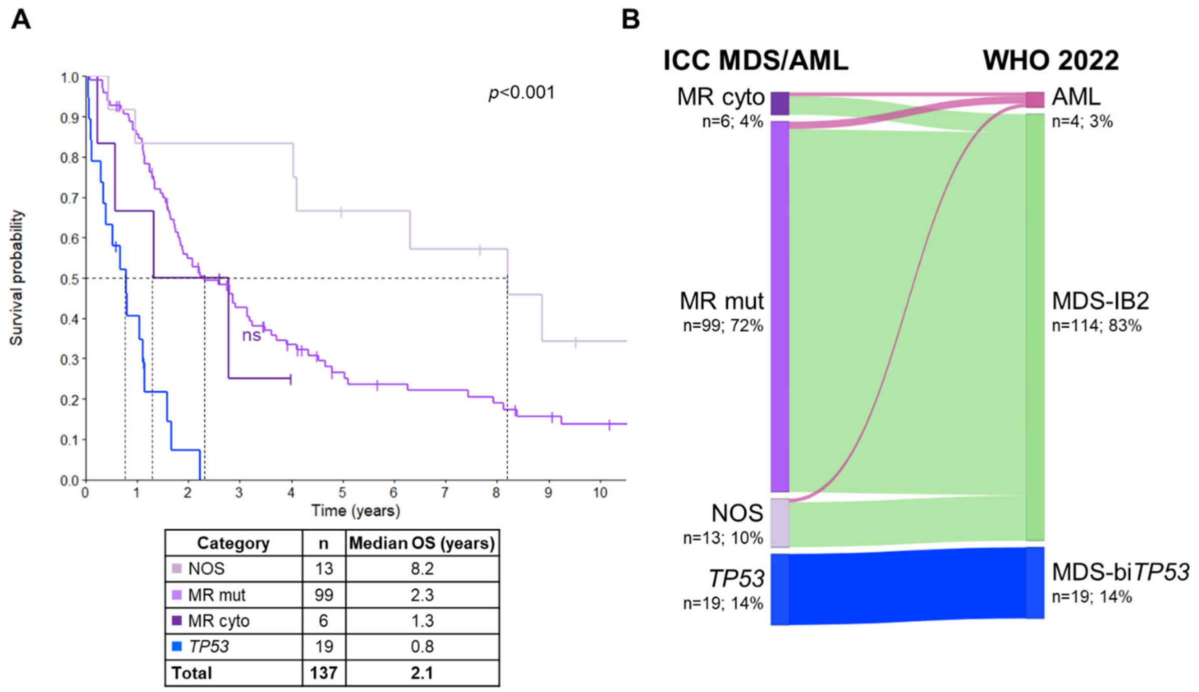

Category	n	Median OS (years)
NOS	13	8.2
MR mut	99	2.3
MR cyto	6	1.3
TP53	19	0.8
**Total**	**137**	**2.1**

Supplementary Figures S1: Survival and subgroups of MDS/AML overlap patients. (A) Overall survival (OS) of the MDS/AML cohort (n=137) according to ICC subgroups. **(B)** Relationship of MDS/AML subgroups and corresponding WHO 2022 diagnosis. NOS: not otherwise specified; MR: myelodysplasia-related; mut: gene mutation; cyto: cytogenetic abnormality; *TP53*: mutated *TP53*; ns: not significant.

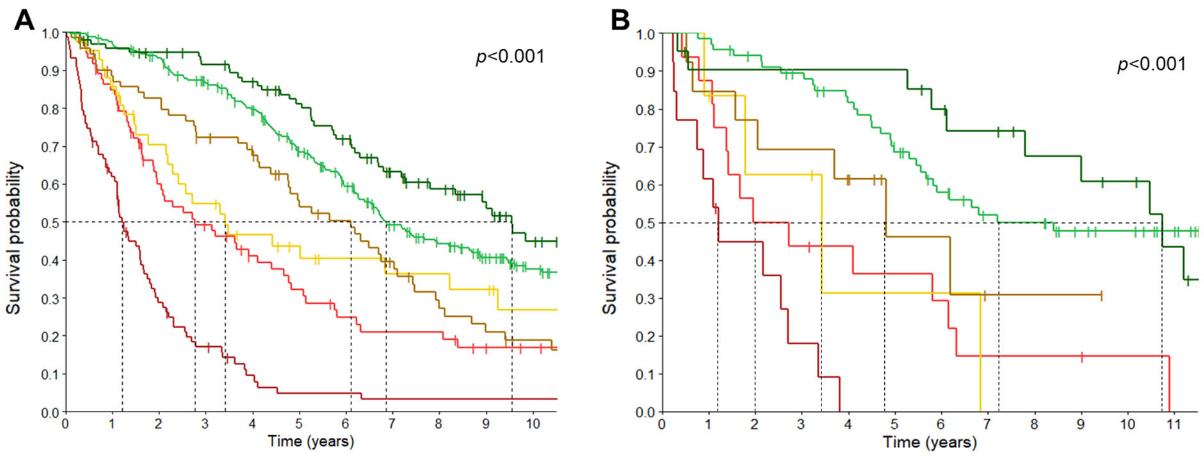

Category	n	Median OS (years)
Very low	97	9.5
Low	254	6.9
Moderate low	71	6.1
Moderate high	42	3.4
High	74	2.8
Very high	88	1.2
**Total**	**626**	**5.7**

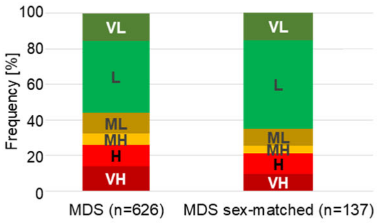

Category	n	Median OS (years)
Very low	21	10.7
Low	68	7.2
Moderate low	13	4.8
Moderate high	6	3.4
High	16	2.0
Very high	13	1.2
**Total**	**137**	**6.1**

Supplementary Figures S2: Survival and frequencies of IPSS-M risk categories of MDS control cohorts. **(A)** Overall survival (OS) of a *bona fide* MDS control cohort ([11]; n=626) according to IPSS-M risk categories. **(B)** OS of a sex-matched MDS cohort (n=137) according to IPSS-M risk categories. VL: very low, L: low, ML: moderate low, MH: moderate high, H: high, VH: very high.

References

1. Schoch C, Schnittger S, Bursch S, Gerstner D, Hochhaus A, Berger U, et al. Comparison of chromosome banding analysis, interphase- and hypermetaphase-FISH, qualitative and quantitative PCR for diagnosis and for follow-up in chronic myeloid leukemia: a study on 350 cases. Leukemia. 2002;16(1):53-9.
2. Haferlach T, Kern W, Schoch C, Hiddemann W, Sauerland MC. Morphologic dysplasia in acute myeloid leukemia: importance of granulocytic dysplasia. J Clin Oncol. 2003;21(15):3004-5.
3. Kern W, Voskova D, Schoch C, Hiddemann W, Schnittger S, Haferlach T. Determination of relapse risk based on assessment of minimal residual disease during complete remission by multiparameter flow cytometry in unselected patients with acute myeloid leukemia. Blood. 2004;104(10):3078-85.
4. Höllein A, Twardziok SO, Walter W, Hutter S, Baer C, Hernandez-Sanchez JM, et al. The combination of WGS and RNA-Seq is superior to conventional diagnostic tests in multiple myeloma: Ready for prime time? Cancer Genet. 2020;242:15-24.
5. Stengel A, Baer C, Walter W, Meggendorfer M, Kern W, Haferlach T, et al. Mutational patterns and their correlation to CHIP-related mutations and age in hematological malignancies. Blood Adv. 2021;5(21):4426-34.
6. Yang H, Chen G, Lima L, Fang H, Jimenez L, Li M, et al. HadoopCNV: A dynamic programming imputation algorithm to detect copy number variants from sequencing data. bioRxiv. 2017:124339.
7. Stengel A, Shahswar R, Haferlach T, Walter W, Hutter S, Meggendorfer M, et al. Whole transcriptome sequencing detects a large number of novel fusion transcripts in patients with AML and MDS. Blood Adv. 2020;4(21):5393-401.

8. Chen X, Schulz-Trieglaff O, Shaw R, Barnes B, Schlesinger F, Källberg M, et al. Manta: rapid detection of structural variants and indels for germline and cancer sequencing applications. Bioinformatics. 2016;32(8):1220-2.

9. Uhrig S, Ellermann J, Walther T, Burkhardt P, Fröhlich M, Hutter B, et al. Accurate and efficient detection of gene fusions from RNA sequencing data. Genome Research. 2021;31(3):448-60.

10. Haas BJ, Dobin A, Li B, Stransky N, Pochet N, Regev A. Accuracy assessment of fusion transcript detection via read-mapping and de novo fusion transcript assembly-based methods. Genome Biology. 2019;20(1):213.

11. Baer C, Huber S, Hutter S, Meggendorfer M, Nadarajah N, Walter W, et al. Risk prediction in MDS: independent validation of the IPSS-M-ready for routine? Leukemia. 2023;doi:10.1038/s41375-023-01831-1.
